# High capacity clinical SARS-CoV-2 molecular testing using combinatorial pooling

**DOI:** 10.1038/s43856-024-00531-w

**Published:** 2024-06-19

**Authors:** Shosh Zismanov, Bar Shalem, Yulia Margolin-Miller, Dalia Rosin-Grunewald, Roy Adar, Ayelet Keren-Naus, Doron Amichay, Anat Ben-Dor, Yonat Shemer-Avni, Angel Porgador, Noam Shental, Tomer Hertz

**Affiliations:** 1 Department of Microbiology and Immunology, Faculty of Health Sciences, Ben-Gurion University of the Negev, Beer-Sheva, Israel; 2 National Institute of Biotechnology in the Negev, Ben-Gurion University of the Negev, Beer-Sheva, Israel; 3 Department of Computer Science, Bar-Ilan University, Ramat Gan, Israel; 4 Poold Diagnostics ltd., Beer-Sheva, Israel; 5 Laboratory of Virology Services, Soroka University Medical Center, Beer-Sheva, Israel; 6 Central Laboratory, Clalit Health Services, Tel Aviv, Israel; 7 Department of Clinical Biochemistry and Pharmacology, Faculty of Health Sciences, Ben Gurion University of the Negev, Beer-Sheva, Israel; 8 Department of Computer Science, The Open University of Israel, Ra’anana, Israel; 9 Fred Hutch Cancer Research Center, Seattle, WA USA

**Keywords:** Diagnostic markers, Health care, Biomarkers

## Abstract

**Background:**

The SARS-CoV-2 pandemic led to unprecedented testing demands, causing major testing delays globally. One strategy used for increasing testing capacity was pooled-testing, using a two-stage technique first introduced during WWII. However, such traditional pooled testing was used in practice only when positivity rates were below 2%.

**Methods:**

Here we report the development, validation and clinical application of P-BEST - a single-stage pooled-testing strategy that was approved for clinical use in Israel.

**Results:**

P-BEST is clinically validated using 3636 side-by-side tests and is able to correctly detect all positive samples and accurately estimate their Ct value. Following regulatory approval by the Israeli Ministry of Health, P-BEST was used in 2021 to clinically test 837,138 samples using 270,095 PCR tests - a 3.1fold reduction in the number of tests. This period includes the Alpha and Delta waves, when positivity rates exceeded 10%, rendering traditional pooling non-practical. We also describe a tablet-based solution that allows performing manual single-stage pooling in settings where liquid dispensing robots are not available.

**Conclusions:**

Our data provides a proof-of-concept for large-scale clinical implementation of single-stage pooled-testing for continuous surveillance of multiple pathogens with reduced test costs, and as an important tool for increasing testing efficiency during pandemic outbreaks.

## Plain language summary

Testing samples for SARS-CoV-2 is usually done on one sample at a time. However, the unprecedented demand for testing during the COVID-19 pandemic led to the adoption of pooled testing strategies, where samples are combined before being tested. This strategy requires two rounds: first, each pool of samples is tested, and then a second testing round is performed on individual samples from positive pools. We developed and implemented a pooling method for SARS-CoV-2 that requires a single round of testing, thus enabling the shorter turnaround times required during a pandemic. The method was approved for clinical use in Israel and was used to successfully test 837,138 clinical samples using fewer than a third of the tests usually required. Our study provides a blueprint for rapid implementation of efficient high-throughput testing in future pandemics.

## Abstract (web summary)

Zismanov, Shalem, Margolin-Miller et al. present the validation and clinical rollout of P-BEST, a combinatorial single-stage pooling method used for mass testing during the SARS-CoV-2 pandemic in Israel. The analysis is a proof-of-concept for the feasibility of using combinatorial pooling solutions for mass testing in clinical settings.

## Introduction

The SARS-CoV-2 pandemic spread rapidly worldwide leading to over 608 million documented infections and 6.5 million deaths^1^. A variety of different mitigation measures were used to control the spread of SARS-CoV-2, which included lockdowns, school closures, travel restrictions, quarantines, diagnostic testing, and vaccination. Until the rise of the Omicron BA.1 variant, community testing for SARS-CoV-2 was utilized by most countries around the world. To date, over 1.5×10^10^ PCR tests have been performed throughout the world, leading to continuous shortages of test reagents, and substantial delays in test turnaround times^2^.

Israel was one of the first countries that offered government funded PCR-based testing to all its residents, rapidly building a network of HMO laboratories and private laboratories to increase testing capacity. To date, a total of 45.5 million PCR tests were conducted in Israel, making it one of the leading countries in COVID-19 tests per capita^2^ (Supplementary Fig. 1). As part of Israel’s strategy for COVID-19 testing, the Israeli ministry of health authorized the use of pooled testing methods, in the spring of 2020. This included the use of traditional Dorfman pooling^3^, which was also approved in other countries^4^, and also P-BEST – a combinatorial single-stage pooling method^5^.

**Fig. 1:**
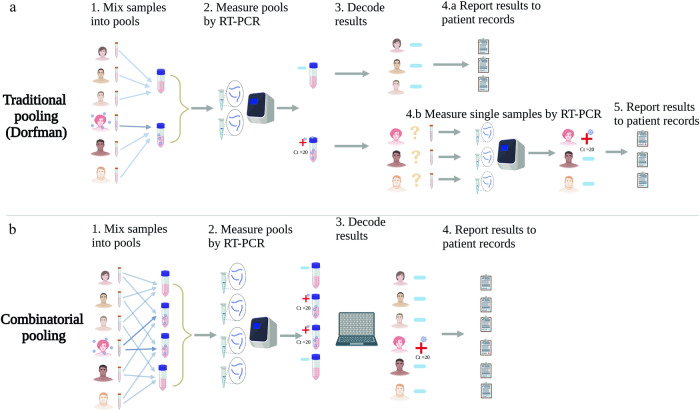
Two-stage pooling vs. single-stage pooling. **a** In traditional two-stage Dorfman pooling, each sample is added to a single pool. Samples belonging to negative poos are classified as “negative” and results can be reported after a single round of testing. Positive pools indicate that one or more of the pooled samples are positive, thus all corresponding samples are re-tested individually and only then, results can be reported. **b** In combinatorial single-stage pooling, each sample is added to several pools, according to a specific pooling design. A decoding algorithm is used to detect all positive samples and their Ct value after a single round of testing.

In contrast to Dorfman pooling, which requires a second round of individual testing of samples that belong to positive pools (Fig. 1a), P-BEST provides diagnostic results within a single round of testing (Fig. 1b). Each pool contains multiple samples and each sample is included in multiple pools, such that the set of measurements across all pools corresponds to a unique set of positive samples and is therefore sufficient to detect these positive samples and their corresponding Ct values after a single round of testing. The approach uses a set of pooling designs that are each optimized for detecting all positive samples up to a specific positivity rate, and as positivity rates change, the specific pooling design used changes accordingly.

Here we present the validation and clinical rollout of P-BEST. To the best of our knowledge, P-BEST was the first combinatorial single-stage pooling method used for mass testing during the SARS-CoV-2 pandemic. P-BEST was validated using a set of 3636 samples were tested side-by-side with individual testing. To facilitate clinical usage of P-BEST a software product (Pooldi) that provides a user-friendly interface of P-BEST was designed and developed by a startup company funded by a grant from the Israel Innovation authority. Here we analyze the clinical use of P-BEST in three large clinical labs during the period of January 2021 to January 2022. Within this period, a total of 837,138 samples were clinically tested using P-BEST using 270,095 PCR reactions, a reduction of 68% in the number of tests performed leading to savings of over 10 million USD in testing reagents alone. Our analysis provides proof-of-concept for the feasibility of using combinatorial pooling solutions for mass testing in clinical settings.

## Methods

### Clinical samples

SARS-CoV-2 clinical Nasopharyngeal swab samples were collected at two public labs and one private lab in Israel. All labs were approved by the Israeli Ministry of Health (MOH) for SARS-CoV-2 molecular diagnostic testing. Upon arrival at the labs, samples were neutralized by incubation at 70 °C for 30 minutes per guidelines by the MOH.

#### Side-by-side validation

For P-BEST clinical validation 3636 samples were used in side-by-side experiments as follows. Each lab tested the samples individually using their standard diagnostic testing protocol, and leftover sample material was subsequently used by P-BEST. In general, P-BEST runs were performed 24–48 hours after standard testing, therefore, in most cases, positive samples were re-tested as singles to provide an accurate Ct value and also to account for viral RNA degradation over time.

#### Clinical testing

Following MOH approval, a total of 837,138 samples were tested with the P-BEST system. Samples classified “positive” or “negative” were reported to medical records, while undecided samples were classified as “suspected” and were individually retested and subsequently reported.

Validation experiments were defined and conducted by the MOH. All de-identified samples used for these experiments were residual samples provided directly by the MOH. This study was exempt from IRB approval and from informed consent as per MOH guidelines. Clinical testing was performed by MOH approved labs and only aggregate data on Ct values of pooled samples were used in the current study.

### Sample pooling

Pools were prepared using a liquid-handling robot (a liquid handling Tecan Freedom Evo 200). To reduce contamination and increase testing efficiency pooling was performed using the original swab collection sample tubes (‘source tubes’) without removing the swabs. Pooling was performed into 5 ml empty tubes (‘pooling tubes’) or 96-well deepwell plates. Source and pooling tubes were placed onto racks, each containing up to 16 samples. The pooling process included automated barcode scanning of the source and pooled samples, allowing automated decoding of the test results. Liquid dispensing parameters were optimized to prevent any sample carryover and to reduce pipetting time.

### Pooling designs

A variety of pooling designs were used for clinical testing (Table 1). Pooling designs were based on pseudo random matrices shown to achieve optimal decoding (Shalem et al. in preparation). Each design was optimized for a specific sample set size ranging from 80 to 384 samples, and for a specific maximal disease prevalence (1%-10%). Designs were tailored per labs’ requirements for small (80 or 96), medium (188) or large ( ~ 384) number of samples. Based on the prevalence of SARS-CoV-2 in Israel during this period several pooling designs were used for clinical testing offering efficiency gains ranging from x1.5-x8.

**Table 1:** P-BEST designs used in clinical settings

Pooling Design (#samples to #pools)	Efficiency	Recommended positivity rate	#samples per pool	Pools per sample	Run time
80 to 10	x8	1%	24	3	15
96 to 25	x3.8	2%	19–20	5-6	24
384 to 94	x4.1	2%	28-29	6-7	55
282 to 94	x3	4%	12	4	45
96 to 46	x2.1	6%	10-11	4-5	28
188 to 94	x2	6%	8	4	25
372 to 186	x2	6%	8	4	55
282 to 188	x1.5	10%	6	4	50

The number of samples and pools in each applied design and their efficiency (i.e., the ratio between them). Recommended positivity rate corresponds to the rate below which the number of “suspected” samples reported by the method is negligible. The run time corresponds to total pipetting time for sample pooling using a Tecan robot.

### Pooled sample testing pipeline

To enable minimally trained lab operators to utilize P-BEST, we developed a user-friendly software product that automates the pooling process. The lab operator selects (a) a specific pooling design based on the current disease prevalence, (b) the specific diagnostic kits used, and (c) the number of samples tested. The user is provided with easy-to-use instructions on how to load source and pool tubes or plates onto the liquid handling robot worktable, after which sample pooling is automatically performed using the liquid handler. Following testing the pools, PCR results are decoded and results are sent to medical records.

### SARS-CoV-2 diagnostic testing

Nucleic acids were extracted from 350ul of sample using the STARMag 2019-nCoV kit (Seegene, CA, USA) on a liquid-dispensing robot (STARlet Hamilton, USA). Elution volume was 100ul. 8 ul of the extracted nucleic acids were taken for cDNA preparation and quantitative reverse transcription PCR (qRT-PCR)–based amplification. qRT-PCR was performed using either of the following clinically approved kits: (a) Allplex 2019-nCoV detection kit (Seegene, CA, USA) which identifies three SARS-CoV-2 genes: E, RdRP, and N; (b) Allplex SARS-CoV-2 Assay (Seegene, CA, USA) which identifies four SARS-CoV-2 genes: E, RdRP, N and S. These assays were performed on a Biorad CFX real-time PCR detection system.

Some of the samples were analyzed on a Panther instrument (Hologic, Inc., San Diego, CA). 500 ul of the sample was taken for RNA isolation, purification, and qualitative detection of SARS-CoV-2, using the Aptima® SARS-CoV-2 assay (Hologic, Inc., San Diego, CA).

### P-BEST decoding algorithm

#### Decoding

P-BEST finds a sparse solution that best fits the measured viral load across pools, thus providing an estimate for the Ct value of each detected sample. In brief, pools whose Ct values < 40 are converted to viral load counts as, where in case of the Seegene kit, was based on the kit’s most sensitive gene, i.e., the N-gene. We then seek the sparsest set of samples that maximizes the likelihood of the pools’ measurements, provided the pooling design and a measurements noise model. For the latter noise model we have experimentally estimated the probability of a false negative measurement as a function of Ct. The set of detected samples and those with zero viral load are provided to the classification module as explained below.

#### Sample classification

First, all samples whose estimated viral load is zero are classified as “negative”, i.e., are COVID-free and do not require retesting. All other non-negative samples are classified as “positive” or as “suspected”. To classify a sample as “positive”, i.e., reported as COVID-positive, a sample should meet the following two criteria: (a) All of its pools should be “strong positive” (i.e., N gene Ct ≤ 36). (b) There exists at least one such “strong positive” pool that contains this sample and does not contain any of the other detected non-negative samples. These criteria are quite stringent and were required to avoid false positive detections. Samples that do not meet these criteria are classified as “suspected” and require re-testing. Specifically, this happens in three cases: (a) The sample belongs to a pool that was invalidated (i.e., the internal control was negative). (b) The sample belongs to a pool which is negative, inconclusive (i.e., some of the genes are not detected) or “weak positive” (i.e., N gene Ct > 36). (c) All of the pools of a specific sample contain other samples that were detected as non-negative.

**Fig. 2:**
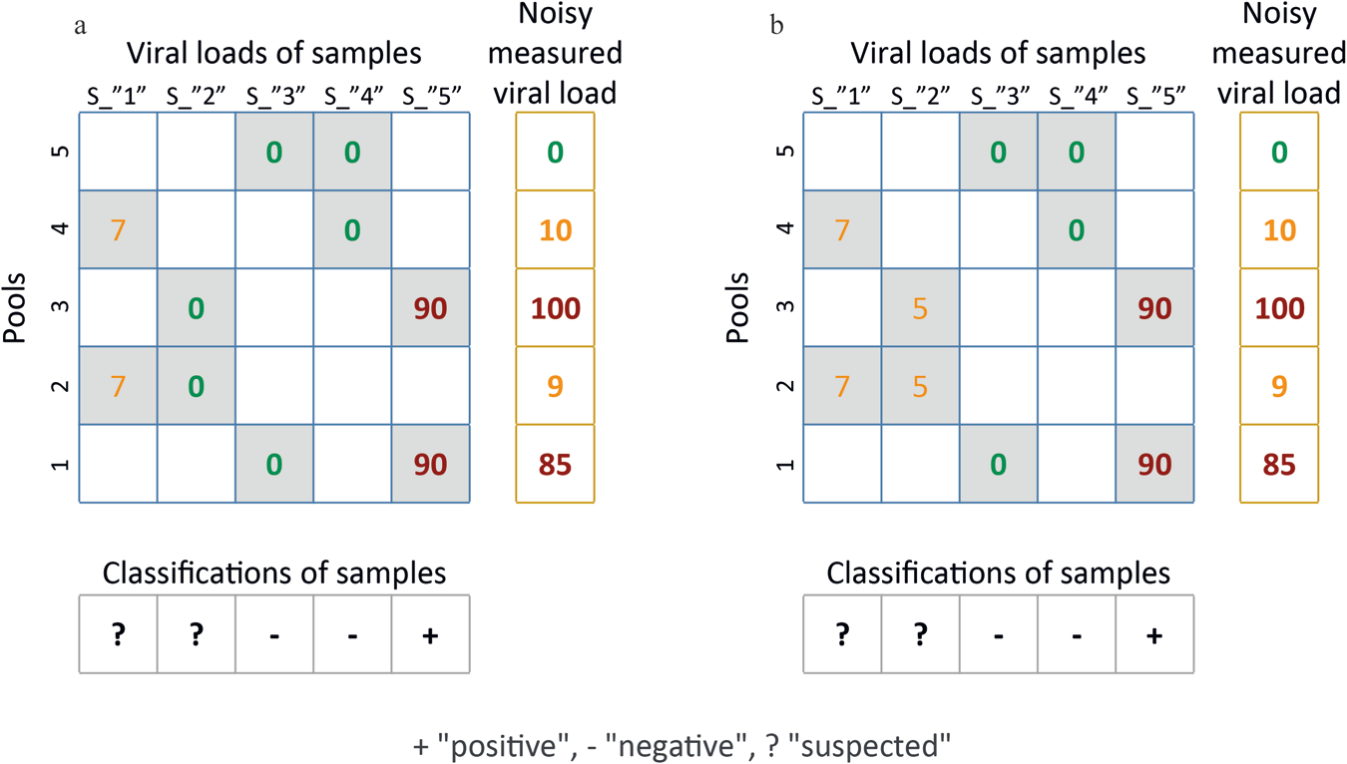
Classification example. **a** A schematic design of five samples and five pools, their viral load measurements, and the classification of each sample as “positive” ( + ), “negative” (-) or “suspected” (?). The numbers in gray shaded squares correspond to the true, although unknown viral load of each sample. **b** The same case as in panel A, with S2 as a weak positive sample.

Figure 2a presents examples of classification scenarios. In the interest of clarity, we present the case of five samples (S_1_-S_5_) being measured in five pools, although in practice the number of pools is always much smaller than the number of samples. Gray shaded squares denote the samples in each pool, e.g., pool #1 in Fig. 2a comprises S_3_ and S_4_. The number inside a gray shaded square corresponds to the true, yet unknown, viral load of the sample. The measured viral loads of each pool should correspond to the sum of the viral loads of its samples, yet are subject to measurement noise. In this example, pools whose viral loads are lower than 20 are considered “weak positive”. The solution to the optimization problem would detect two “negative” samples (S_3_, S_4_), and would assign non-negative values to S_1_, S_2_ and S_5_. The latter samples are classified as detailed below.

S_5_ is classified as “positive” since (a) both its pools have a high enough viral load, and (b) pool #5 is unique to S_5_ and does not contain the other detected samples, S_1_ and S_2_.

Both pools containing S_1_ are “weak positive” pools, and, therefore, it is classified as “suspected”. Similarly, S_2_ is classified as “suspected” since one of its pools (pool #4) is “weak positive”. Subsequent re-testing of single samples is deemed to find S_2_ as “negative” in this case, yet a different scenario is also plausible. Figure 2b is identical to Fig. 2a except for the fact that S_2_ has a low viral load rather than being negative, i.e., two different scenarios for S_2_ share the same pooling measurements. Hence, since measurement noise cannot discern between a negative and a weak positive S_2_, it is classified as “suspected”.

### Statistics

Comparisons between groups were performed using the Wilcoxon rank-sum test.

### Reporting summary

Further information on research design is available in the Nature Portfolio Reporting Summary linked to this article.

## Results

### Clinical validation of P-BEST

To validate P-BEST and obtain regulatory approval for its use in clinical SARS-CoV-2 diagnostic testing in Israel, we followed guidelines outlined by the Israeli MOH, which required side-by-side validation studies to be conducted in each lab independently. We conducted a set of 35 side-by-side experiments comparing P-BEST to testing each sample individually. A total of 3,636 samples, collected between 24/11/2020 and 07/02/2021, were tested during the validation stage (Supplementary Table 1). All samples were tested individually 24-48 hours prior to P-BEST pooling, and most positive samples were also individually re-tested in tandem with P-BEST to account for potential signal decay of stored samples. We demonstrated that P-BEST correctly classified all samples with zero false positives and a single false negative, and correctly estimated the Ct values of positive samples (Table 2) leading to regulatory approval for clinical diagnostic testing by the Israeli ministry of health (MOH). Pooldi was then installed in 3 major diagnostic labs in Israel, and routinely used for clinical SARS-CoV-2 diagnostic testing starting in January 2021.

**Table 2:** Evaluating P-BEST classification performance using side-by-side testing

Single classification	#Samples	P-BEST classification				
		#“Positive”	#“Negative”	#“Suspected”		
				*Invalidated pool*	*Weak pool*	*Overcrowding*
Positive (Ct < 35)	141	97	1	6	16	21
Weak positive (Ct > 35)	13	0	5	-	8	-
Negative	3482	0	3383	4	75	20

Each sample was tested individually and then using the P-BEST pooling. P-BEST’s classifications of samples as “positive”, “negative” or “suspected” (i.e., requiring retesting) are presented. “Suspected” calls are further classified into 3 categories: (i) *invalidated pool*: cases in which the detected sample participated in a pool whose PCR measurement was invalidated. (ii) *weak pool*: corresponds to cases in which the sample was part of a pool whose PCR was negative or of Ct>36. (iii) ‘*overcrowding*’: are cases in which a positive sample shares all of its pools with other positive samples and thus cannot be decisively classified.

### Evaluation of P-BEST calls

Validation experiments were performed using three pooling designs, that were relevant to SARS-CoV-2 prevalence levels in Israel during this period: (a) 96 samples pooled into 25 pools (x3.8 efficiency gain) suitable for up to 2% disease prevalence (19 experiments); (b) 96 samples pooled into 46 pools (x2.1 efficiency gain) suitable for up to 6% disease prevalence (15 experiments); and (c) 372 samples pooled into 186 pools (x2 efficiency gain) suitable for up to 6% disease prevalence (1 experiment). In each experiment the number of positive samples matched or exceeded the maximal prevalence of the pooling design used (Supplementary Table 2). This allowed us to also assess the performance of P-BEST in situations where the actual prevalence may be higher than expected - a scenario which may occur in real-world clinical settings due to batch effects (e.g., an infected family), or to dynamic changes in disease prevalence caused by pandemic waves. Validation was performed following the MOH guidelines for clinical pooled testing, requiring correct detection of all samples with Ct values below 35. A total of 141 positive samples whose Ct was lower than 35 were included in this set (Table 2).

The P-BEST algorithm classifies each sample into one of three categories: (1) “Positive” - a sample from an infected individual; (2) “Negative” - a sample from an un-infected individual; and (3) “Suspected” - samples that require retesting individually due to several types of inconclusive or partial measurements, as detailed below. The criteria defining the “Suspected” category was defined by the clinical laboratories to minimize the number of false positives. P-BEST classified 97 out of the 141 positive samples as “positive”. The remaining 44 positive samples were classified as “suspected”, due to one of the following reasons: (a) Invalidated pools - samples which were included in a pool that was classified as invalid due to technical PCR issues (n = 6). (b) Weak pools - Samples that appeared in at least one pool with high Ct values (n = 16). (c) Overcrowding - Samples included in overcrowded experiments where the actual positivity rate exceeded the maximal positivity rate of the pooling design used (n = 21).

Importantly, there were zero false positive calls, and a single case of a false negative call. This specific positive sample was included in five pools, three of which were negative, one had a Ct value of 39.9 and a fifth pool contained an additional positive sample with a low Ct value. Although the initial Ct of the false negative sample was 32.2 it was not measured in tandem with the pooling experiment, performed 18 hours afterwards, and may have been missed due to sample deterioration. We also analyzed 13 weak positive samples (Ct > 35) which were not required to be detected by P-BEST per the Israeli MOH guidelines. We found that P-BEST classified 8/13 (61%) of these samples as “suspected”. This corresponds to the detection rate of the Seegene SARS-CoV-2 kit for weak samples, as specified by the manufacturer.

### P-BEST provides accurate Ct estimates

**Fig. 3:**
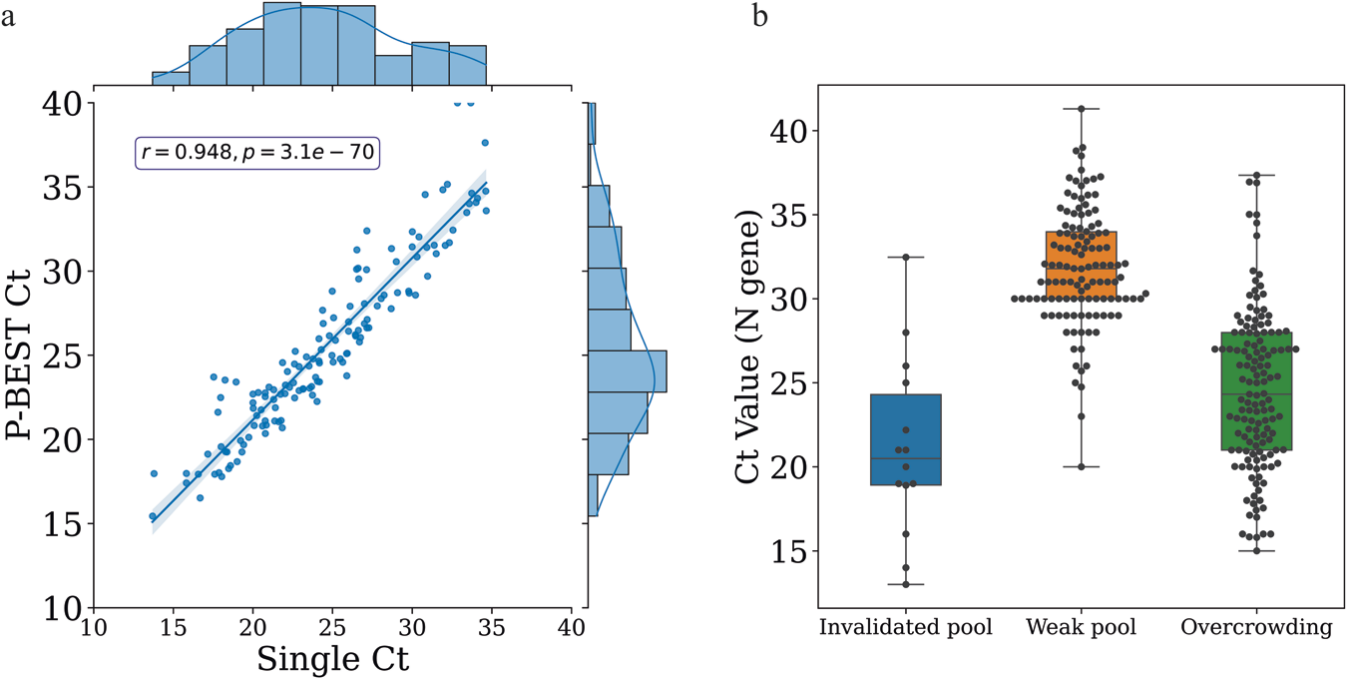
Ct value estimates via P-BEST. **a** A scatter plot of the Ct value of positive samples as measured individually (x-axis) vs. their P-BEST estimated value (y-axis). Shown is the Pearson correlation coefficient. **b** Boxplots of the the Ct value (as measured individually) of samples classified by P-BEST as “suspected”. Samples are divided into 3 groups according to the reason for their “suspected” classification, i.e., *invalidated pools* (blue), *weak pools* (orange), and *overcrowding* (green). Each dot represents the Ct value of a single sample. Black lines represent the median, boxes denote the 25th and 75th percentiles, and error bars represent 1.5 times the interquartile range.

Another advantage of P-BEST is its ability to estimate the Ct value of positive samples. Using positive samples identified by individual testing, we compared the Ct values of the N-gene as measured using the Seegene COVID-19 PCR kit, to the ones estimated by P-BEST. We found that the correlation between the measured and estimated Ct values was r = 0.95 (Pearson correlation, Fig. 3a, Source data underlying the figures are available as Supplementary Data 1). To further analyze the Ct estimates of P-BEST we stratified the Ct values of samples that were classified as “suspected” using the 3 categories defined above (i.e., invalidated pools, weak-pools and overcrowding) and plotted their individual Ct values as measured using the Seegene assay (Fig. 3b, Source data underlying the figures are available as Supplementary Data 2). As expected, we observed that the Ct values of samples classified due to weak pools were indeed significantly higher than those of the two other categories that lead to “suspected” classifications (p < 6×10^−8^ and p < 11×10^−28^, respectively, Wilcoxon ranksum test).

### Using P-BEST for clinical diagnostics

**Fig. 4:**
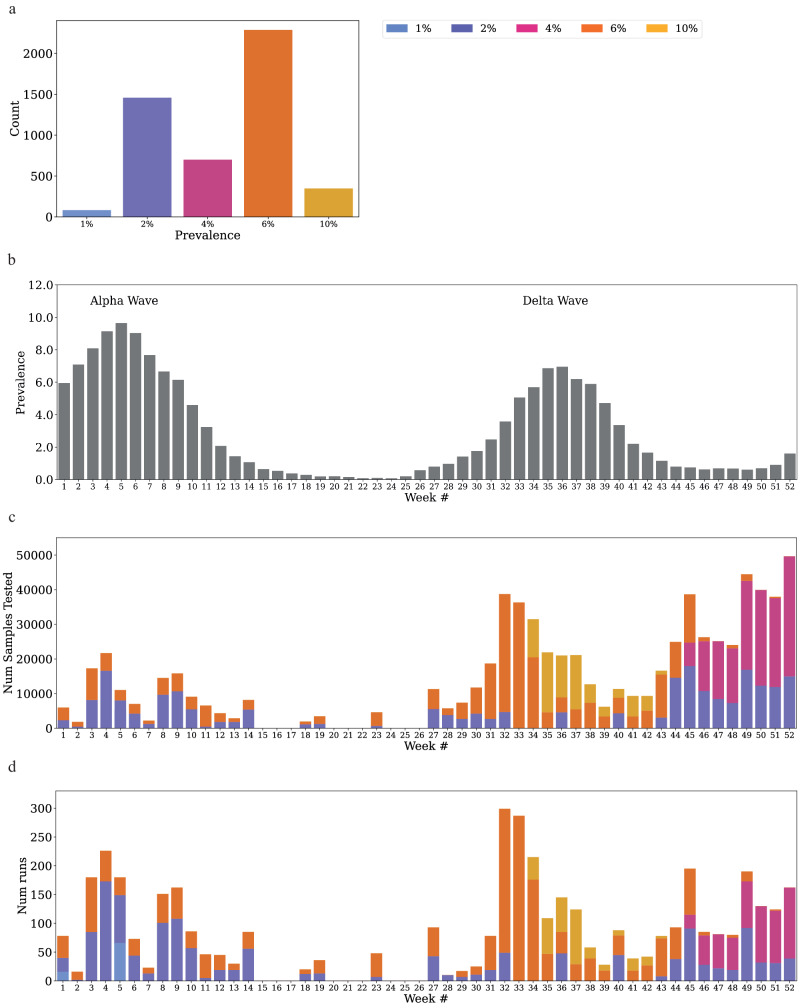
P-BEST in clinical diagnostics during 2021. **a** The number of P-BEST runs performed stratified by pooling design. **b** The average weekly positivity rate in Israel in 2021 based on Israeli MOH published data. **c** Weekly number of samples tested by P-BEST color-coded by pooling design. **d** Weekly number of runs performed using P-BEST color-coded by pooling design. The same as C for the number of runs.

Following the completion of the validation studies, P-BEST was approved for clinical use in Israel by the Israeli MOH. During the 13 months of clinical operation (January 2021 to January 2022), a total of 837,138 samples were tested with P-BEST using 270,095 PCR reactions, corresponding to an average efficiency of 3.1 and a reduction of 68% in the number of tests performed. We executed a total of 4874 runs with the following efficiencies: 347 runs with 1.5x efficiency, 2287 runs with 2x efficiency, 700 runs with 3x efficiency, 1458 runs with 4x efficiency and 82 runs with 8x efficiency (Fig. 4a, Source data underlying the figures are available as Supplementary Data 3). Throughout this period, which included the Alpha and Delta waves, positivity rates fluctuated ranging from 0.8% to 10.2%, (Fig. 4b, Source data underlying the figures are available as Supplementary Data 4). The specific pooling designs used were selected based on the positivity rate per week (Fig. 4c, d, Source data underlying the figures are available as Supplementary Data 5 and 6). During the Alpha wave most of the tests performed used the 2% or 6% designs, even when positivity rates were higher, thanks to a pre-testing screening procedure performed by some of the clinical laboratories. In particular, samples were classified into ‘high’ and ‘low’ risk of positivity, based on de-identified demographic information, and only ‘low’ risk samples were pooled using P-BEST. For example, samples from a testing program called Magen Avot – which included all nursing care homes in Israel, were classified as low-risk due to the tight restrictions imposed in these nursing homes, coupled with PCR-based testing twice every week – which prevented major community spread within these homes. While this strategy effectively reduced the positivity rates by about 2-4%, rates were still higher than those suitable for traditional Dorfman pooling. During the peak of the Delta wave, a 10% pooling design was used in combination with the pre-screening testing approach described above, allowing labs to continue pooling samples even when de facto rates exceeded 10% (Fig. 4c, d, Source data underlying the figures are available as Supplementary Data 5 and 6).

### High capacity pooling during the tail of the Delta wave

While positivity rates at the end of the Alpha wave in Israel were very low ( < 0.8%, Fig. 4b, Source data underlying the figures are available as Supplementary Data 4), resulting in a sharp reduction in the total number of PCR tests performed (Supplementary Fig. 2), the Delta wave declined more gradually with an average positivity rate of ~1% (Fig. 4b, Source data underlying the figures are available as Supplementary Data 4). During November to December 2021, the labs converted their entire testing pipeline into a pooling pipeline using P-BEST, and a total of 210,090 samples were tested using pooling designs of 1%-4%. The number of PCR reactions used to test these samples in total was 62,040 tests, a reduction of 70% in testing reagents used, which translated to considerable financial savings for the labs.

### Samples requiring retest are mainly due to improper use of P-BEST

During the Alpha wave, 6% of the samples tested were classified as “suspected” by P-BEST, thus requiring re-testing (see Methods). We found that “suspected” calls were not uniformly distributed across runs, with 60% of them appearing in only 7% of the runs. These runs were “overcrowded” runs in which the effective positivity rate exceeded the recommended rate of the pooling design used for testing. This was mostly due to human error as samples whose pre-screening classification was “high-risk” were tested using P-BEST, rather than being tested as individual samples. Excluding these runs, the empirical efficiency during the Alpha wave reached 3.1x corresponding to a reduction of 68% in the number of tests. We note that during the Delta wave, following additional training of lab personnel and sufficient hands-on experience of using the software, the number of “suspected” samples that required retesting was substantially reduced.

### Adapting combinatorial pooling to low-resource laboratories

**Fig. 5:**
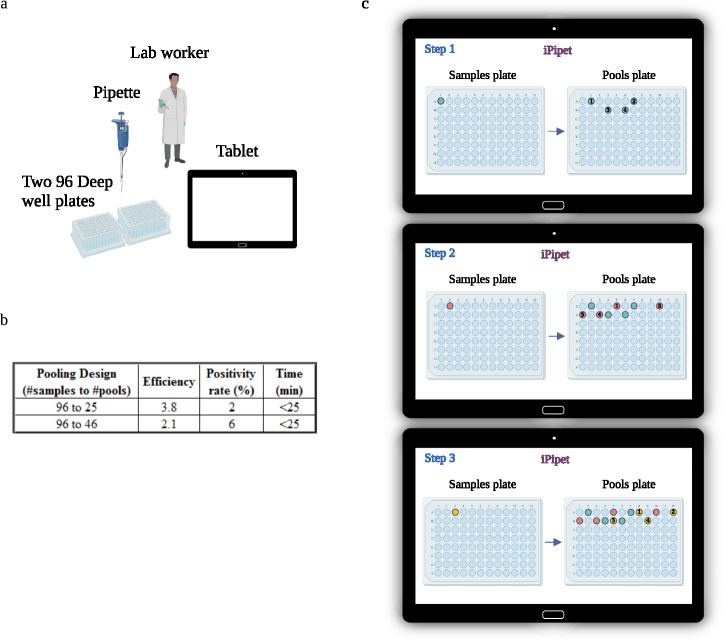
Tablet-directed pooling. **a** Description of the equipment required for manual pooling. **b** The number of samples and pools in each manual design and their efficiency. Recommended positivity rate corresponds to the rate below which the number of “suspected” samples reported by the method is negligible. The run time corresponds to total pipetting time for sample pooling using a manual pipette. **c** An illustration of the first three iPipet^6^ pipetting operations using two 96-well plates on top of a standard tablet.

A key challenge in implementing combinatorial pooling is the need to obtain a costly liquid dispensing robot, which also requires continuous technical support. While such robots have become common in many western-world laboratories, they are not widely used in many low- and mid-income countries. We therefore sought to adapt our pooling method for use in such settings. Building upon a tablet-directed pooling solution^6^ we developed a version of our pooling method for manual combinatorial pooling which requires minimal laboratory equipment (Fig. 5a). Since all pipetting operations are performed by a laboratory technician, we used small scale pooling designs that pool 96 samples into 25 or 46 pools and take less than 25 minutes (Fig. 5b). To make pipetting more user-friendly, we re-ordered the pipetting operations such that the required movements are always from left to right, and dispensing is more regular (e.g., two dispensing operations per row of pools) (Fig. 5c). Decoding was performed using an online cloud-based server onto which the technician could load a PCR result file. We also extended our decoding algorithm to identify manual pipetting errors (e.g., erroneous pipetting into a wrong well), usage of an incorrect pooling matrix, etc. Basically, such errors are identified by detecting discordance between the measured viral load of a pool and the estimated Ct values of its samples. We tested our manual pooling method with two SARS-CoV-2 PCR kits developed in India, in collaboration with a local lab. Pilot studies demonstrated that all positive samples were properly detected using our approach (Table 3).

**Table 3:** Results of pilot experiments using a tablet-based manual implementation of P-BEST

# samples	# pools	# positives	# detected true positives	# suspected that were indeed positive	# suspected that were negative
96	46	6	6	1	0
96	46	6	5	1	3
96	25	2	2	0	0
96	25	2	2	0	0

Two manual pooling designs were tested – (i) using 46 pools to test 96 samples (6% positivity rate); and (ii) using 25 pools to test 96 samples (2% positivity rates). Each design was tested twice by a laboratory in India. Results were analyzed using a cloud-based implementation of the P-BEST method.

## Discussion

The SARS-CoV-2 pandemic led to a massive expansion of molecular testing capacity around the world. Despite this, in many countries turnaround times for PCR testing were very slow, both due to limited testing capacity and shortages of testing reagents. While some countries such as the USA, the UK, and Uganda authorized two-stage traditional pooled testing^7–9^, pooling was not widely used to increase testing capacity and reduce costs. To the best of our knowledge, Israel was the only country in the world to validate and authorize a combinatorial single-stage pooling method for clinical use, which among other factors allowed it to offer some of the highest testing capacity per-capita in the world^2^.

Several studies reported results using adaptive two-stage group testing, i.e., pooling methods for SARS-CoV-2 testing^3,10,11^. The clinical testing described here utilized the P-BEST (Pooling-Based Efficient SARS-CoV-2 Testing) method, which, as opposed to former methods, is a non-adaptive group testing approach that requires only a single round of testing^5^. Several other papers have since presented analogous non-adaptive approaches^12–16^, yet, to the best of our knowledge, none of these methods were utilized in clinical settings.

We summarized data from 13 months of clinical use of P-BEST for COVID-19 PCR testing. During this period, a total of 837,138 PCR results were processed using P-BEST in 3 large clinical diagnostic laboratories in Israel. Pooled testing was not only performed in periods of low disease prevalence, but also during the Alpha and Delta waves in Israel, including a period where positivity rates exceeded 10%. The use of pooled testing resulted in a reduction of 68% in the number of PCR kits used, allowing labs to increase testing capacity even during infection peaks. The onset of the Omicron wave in January 2022 led to changes in testing guidelines in Israel, and PCR testing was offered only to symptomatic individuals and only after a positive rapid antigen test result. This led to a dramatic reduction in the overall number of PCR tests, as well as to skyrocketing positive PCR tests surpassing 60%. Under these circumstances, it was no longer feasible to utilize pooled testing and labs shifted back to single sample testing.

While P-BEST was developed in early March 2020, and preliminary validation experiments were previously published, the current study reports and analyzes its use in large-scale clinical settings during 2021. These data demonstrate the clinical feasibility of using combinatorial single-stage pooled testing in pandemic settings, and propose that this approach should be further developed as part of pandemic preparedness programs providing an additional important strategy for diagnostic testing. This rapid and effective clinical implementation of P-BEST was possible due to several important factors: first and foremost, the Israeli MOH actively sought innovative ideas for increasing testing capacity, and provided a well-defined and rapid regulatory pathway for clinical validation of new testing methods. Second, a startup company funded by a grant from the Israeli Innovation Authority was founded to develop a user-friendly software product that implemented P-BEST, allowing minimally trained laboratory technicians to easily use the method, and also integrating in medical record systems. Finally, a close collaboration with the Clalit Healthcare virology labs was critical for gaining hands-on experience of the lab testing workflows, and for obtaining critical feedback regarding the usability and requirements of the software product implementing P-BEST.

Another critical factor for clinical implementation was reducing overall test-time. Since pooling introduces an additional pre-processing step, it may increase testing time thereby increasing labs’ time-to-result, which was an important performance measure utilized by the Israeli MOH during the pandemic. To reduce test turnaround time, we developed an optimization procedure that minimizes the number of pipetting steps by the liquid handling robot. This enabled testing more than 5000 samples per day on a single Tecan Freedom EVO 200 robot. In one of the labs, the overall pooled testing capacity was 15,000 samples per day.

P-BEST classification calls were tuned towards reducing false positive calls. For example, a sample of very low Ct would be called “suspected” if one of its pools was invalidated due to technical issues. In clinical practice, 3.4% of samples were classified as “suspected”, and most of these (56%) were due to ‘weak pools’, i.e., pools with Ct > 36 or even negative pools. An additional 41% were classified as “suspected” due to ‘overcrowding’. It should be noted that these criteria are utilized as post-processing decisions and may be easily altered in different testing scenarios. In practice, 70% of the “suspected” calls (16,613) originated from 12.6% (n = 613) of the total number of pooling runs (n = 4874). Similarly, the retest rate in the validation stage was 150/3636 (4.1%) and the majority of these “suspected” calls were due to “overcrowded” experiments in which the frequency of positive samples included in the test set was intentionally larger than the one that the pooling design used was optimized for, or due to “dropped pools” due to PCR technical issues unrelated to the pooling process. These experiments were performed in order to evaluate the degradation in performance that would occur due to overcrowding, or PCR technical issues. Although theoretical guarantees are impractical, our clinical data demonstrate that P-BEST provides reliable diagnostic results, and was adapted for mass diagnostic testing by three major laboratories in Israel.

In general, the performance of P-BEST depends on the limit of detection (LoD) of the kit used for downstream analysis. For example, the LoD of the Seegene PCR kit used in this study, as stated by the FDA approved EUA, corresponds to a Ct of 33^17^, i.e., below which at least 19 of 20 technical repeats would yield a positive result. Clinical data from the validation study in the Soroka University Medical Center virology lab (one of the three labs using P-BEST clinically) demonstrated an effective LoD Ct of 35. This increased sensitivity may be due to the repeated measurements of each sample when using combinatorial pooling. Therefore, clinical use of P-BEST requires calibration to each individual testing kit used, using side-by-side testing of individual and pooled samples. Indeed, this was performed for multiple PCR kits which were in clinical use within these laboratories, and typically took less than a week for each new kit.

Another strategy used in Israel during the pandemic was pod-testing, in which each individual was sampled using two swabs - one that was added into a Pod, i.e., a pool consisting of multiple swabs in a single large tube, and another individual sample was stored in a standard UTM tube. Pods were tested, and every positive pod was then deconvoluted by testing all of the individual tubes that were included in the pod. This strategy was widely used for testing nursing homes and schools, as well as individuals arriving at the airport. Pod sizes ranged from 5-25, depending on the positivity rate at a given time point. While this strategy was highly effective when positivity rates were very low, we found that when positivity rates exceeded 1% - labs effectively ceased testing the pods and resorted to individual sample testing. In contrast, during the Alpha and Delta waves in Israel, we demonstrated that our approach can be effectively used for positivity rates of up to 10% by high-capacity clinical labs. While from a theoretical perspective, optimal Dorfman pooling can reduce the number of tests comparable to the combinatorial pooling approach, in practice Dorfman pooling was not utilized for clinical testing when rates begin to rise, due to the additional labor involved in the large number of retests required using this approach (Supplementary Fig. 3). We note that one advantage of the pod testing approach used in Israel is that since each individual is sampled twice, labs can automatically decide if to use the two-stage pod-testing strategy, or switch to combinatorial pooling when positivity rates rise, without having to modify the sampling scheme used. This is done simply by ignoring the pods, and using combinatorial pooling on the individual sample tubes.

P-BEST can be easily adapted to diverse diagnostic tests, including multiplex testing of several pathogens. The latter case is highly important since it is unique to single-stage group testing and cannot be performed via Dorfman pooling. The de-facto prevalence when performing Dorfman pooling for screening multiple pathogens is the sum across the prevalence of each target in the multiplex test. Therefore, the number of pools whose samples need to be retested may be very large, rendering Dorfman pooling highly inefficient. In contrast, the de-facto prevalence for P-BEST is governed by the maximal prevalence across targets. Hence, P-BEST can be applied for simultaneous detection of carriers of multiple infectious agents, e.g., carriers of SARS-CoV-2 and Flu. Preliminary results demonstrated the ability of P-BEST to correctly identify positive samples of various respiratory viruses (Flu A, Flu B, MPV, PIV, RSV, HRV), using the Allplex™ RV7 Essential Assay (Seegene, Supplementary Data 7). We also correctly identified samples tested using GeneXpert® Xpress multiplex kits for SARS-CoV-2/FLU and for RSV/FluA/FluB using two different pooling designs. (Supplementary Data 8-9). The P-BEST pooling method can be easily applied to other multiplex PCR assays being especially efficient for large scale screening, e.g., for urinary tract infections, gastrointestinal and sexually transmitted infections.

While multiple labs have successfully utilized Dorfman pooling strategies during the pandemic^9,10^, there are three clear advantages of the combinatorial pooling strategy presented here: (1) In combinatorial pooling each sample is added into multiple (3-5) pools and in essence is tested multiple times. Our clinical experience demonstrated that such repeated measurements can counteract the dilution effect due to sample pooling, effectively increasing sensitivity over single-well pooled testing; (2) The clinical utility of combinatorial pooling (but not Dorfman pooling) when positivity rates range between 2-10%; and (3) The ability to use combinatorial pooling for multiplexed diagnostic testing which is now becoming increasingly widespread. On the other hand, it is clear that the simplicity of traditional Dorfman testing, allows its rapid integration into testing pipelines, including labs that do not utilize liquid dispensing robots.

While our data demonstrates high-capacity combinatorial pooled testing, that requires liquid dispensing robots, we also showed that our method can be adapted for use in low-resource settings where robots are not available. Such a solution is important for future pandemics, since shortages in testing reagents would inevitably impact low and middle-income countries. The implementation of a manual combinatorial pooling solution only requires a tablet and a manual pipette, which are readily available worldwide.

In sum, we report a high-throughput application of combinatorial single-stage pooling. To the best of our knowledge, this is the first application of such a method in clinical settings. The large number of clinical tests performed using P-BEST during the pandemic demonstrates the feasibility of implementing combinatorial pooled testing in clinical settings, which may be critically important in future pandemics, and also for conducting large scale screening studies using multiplex PCR kits. As molecular diagnostic testing becomes more prevalent, P-BEST offers a strategy that may allow continuous surveillance for multiple common human pathogens with manageable test costs and using existing testing infrastructure.

### Supplementary information

Peer Review File

Supplementary Information

Description of Additional Supplementary Files

Supplementary Data 1

Supplementary Data 2

Supplementary Data 3

Supplementary Data 4

Supplementary Data 5

Supplementary Data 7

Supplementary Data 6

Supplementary Data 8

Supplementary Data 9

Reporting Summary

## Data Availability

Source data underlying the figures are available as Supplementary Data 1-6.

## Code availability

All analysis code will be available upon request.
