# Supplementary material for: High capacity clinical SARS-CoV-2 molecular testing using combinatorial pooling
Source: Commun Med (Lond). 2024 Jun 19;4:121. doi: 10.1038/s43856-024-00531-w (PMC11187214; doi:10.1038/s43856-024-00531-w)
Supplement: Supplementary file 2 — Supplementary Information [file 43856_2024_531_MOESM2_ESM.pdf]

Title: High capacity clinical SARS-CoV-2 molecular testing using combinatorial pooling

Authors: Shosh Zismanov[1,2]†, Bar Shalem[3]†, Yulia Margolin-Miller[4]†, Dalia Rosin-Grunewald[4], Roy Adar[4], Ayelet Keren-Naus[1,5], Doron Amichay[6,7], Anat Ben-Dor[6], Yonat Shemer-Avni[1,5], Angel Porgador[1,2], Noam Shental[8]*, Tomer Hertz[1,2,9]*

Affiliations:

[1]Department of Microbiology and Immunology, Faculty of Health Sciences, Ben-Gurion University of the Negev, Beer-Sheva, Israel.

[2]National Institute of Biotechnology in the Negev, Ben-Gurion University of the Negev, Beer-Sheva, Israel.

[3]Department of Computer Science, Bar-Ilan University, Ramat Gan, Israel.

[4]Poold Diagnostics ltd.

[5]Laboratory of Virology Services, Soroka University Medical Center, Beer-Sheva, Israel.

[6]Central Laboratory, Clalit Health Services, Tel Aviv, Israel.

[7]Department of Clinical Biochemistry and Pharmacology, Faculty of Health Sciences, Ben Gurion University of the Negev, Beer-Sheva, Israel.

[8]Department of Computer Science, The Open University of Israel, Ra'anana, Israel.

[9]Fred Hutch Cancer Research Center, Seattle, WA, USA

* Corresponding authors. Email: Tomer Hertz, thertz@post.bgu.ac.il, Noam Shental, shental@openu.ac.il

† These authors contributed equally

Supplementary Materials:

Supplementary Figs. 1 to 2

Supplementary Tables 1 to 2

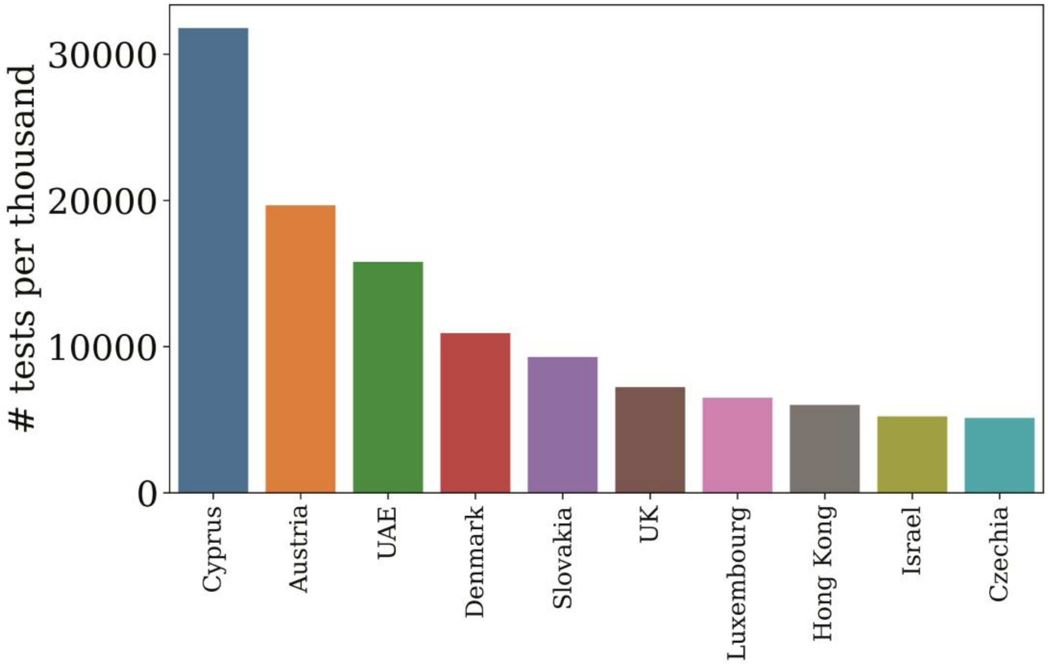

Supplementary Fig. 1: Total PCR tests performed per 1,000 individuals by country - 31.3.2022

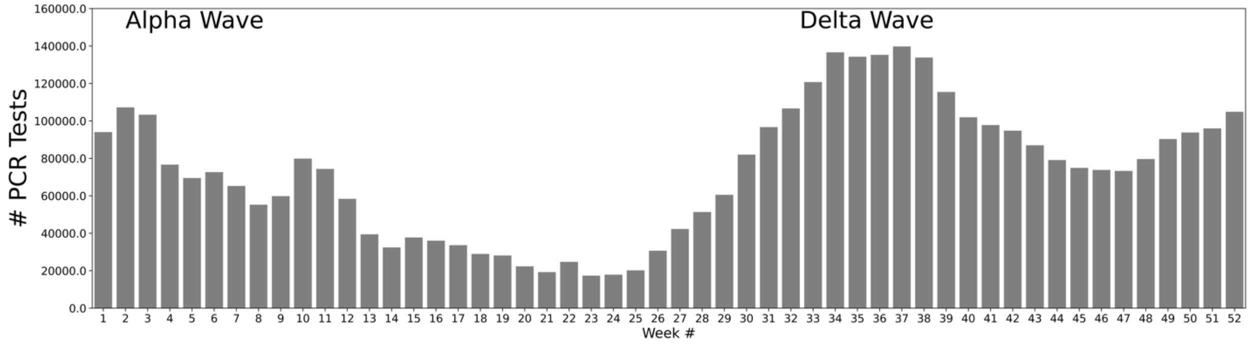

Supplementary Fig. 2: Total number of PCR tests performed by week in Israel during 2021.

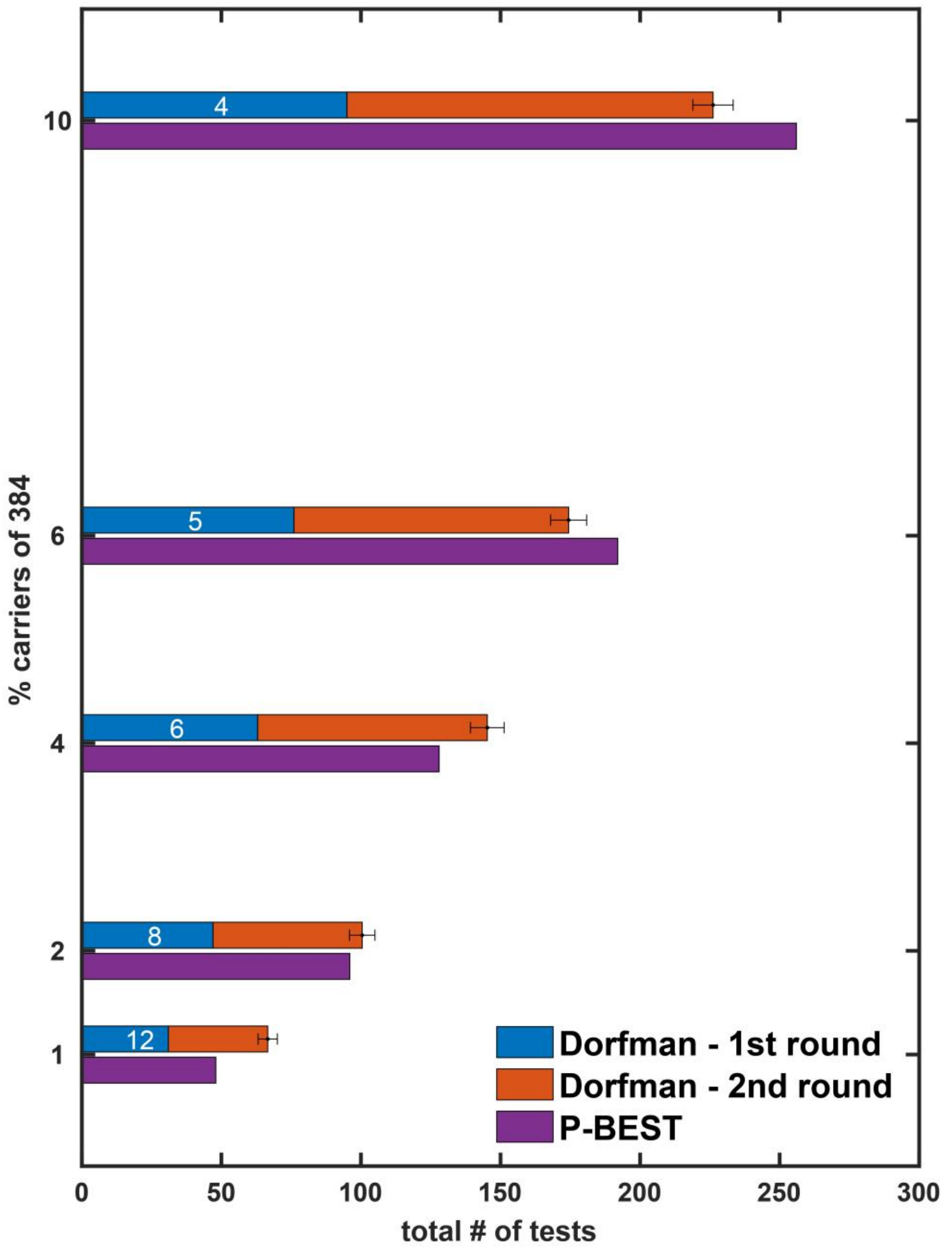

Supplementary Fig. 3: Comparison of the number of tests of the two-stage Dorfman pooling method vs. P-BEST. The total number of tests performed for screening 384 samples via standard two-stage Dorfman pooled-testing and in P-BEST. Shown are data for five positivity rates - 1%, 2%, 4%, 6% and 10%. Dorfman pooling: Shown are the number of tests performed in the first and second rounds of Dorfman pooling (blue and red bars, respectively). The Dorfman pool size (shown inside the blue bar) was optimized to minimize the total number of tests. Dorfman results are based on 10,000 random simulations; error bars

correspond to the standard deviations. P-BEST: The total number of tests performed by P-BEST is, in some cases, higher than the number required by Dorfman, yet the number of tests performed in Dorfman's second stage makes it prohibitive in many realistic scenarios.

Supplementary Table 1: Side by side data

Validation num	#samples	#pools	Date	Positive samples in the run				Negative samples in the run				Weak positive sample in the run			
				# positive	True positive	False negative	True suspected	# negative	False positive	True negative	False suspected	#week positive	Weak true positive	Weak false negative	Weak true suspected
1	96	25	24/11/20	2	0		2	94		94	0				
2	96	25	26/11/20	2	1		1	94		92	2				
3	96	25	26/11/20	2	1	1	0	94		91	3				
4	96	25	26/11/20	2	2		0	93		93	0	1		1	
5	96	25	27/11/20	2	0		2	93		93	0	1			1
6	96	25	12/1/20	2	1		1	94		94	0				
7	96	25	12/1/20	2	2		0	94		94	0				
8	96	25	27/11/20	3	2		1	93		91	2				
9	96	25	27/11/20	3	3		0	93		91	2				
10	96	25	27/11/20	3	2		1	93		93	0				
11	96	25	27/11/20	3	3		0	93		90	3				
12	96	25	12/1/20	3	3		0	93		93	0				
13	96	25	12/1/20	3	2		1	93		91	2				
14	96	25	27/11/20	1	0		1	94		90	4	1		1	
15	96	25	27/11/20	2	1		1	93		91	2	1			1
16	96	25	27/11/20	2	1		1	93		92	1	1		1	
17	96	25	12/1/20	2	1		1	93		90	3	1			1
18	96	25	12/1/20	1	1		0	93		90	3	2		1	1
19	96	25	12/1/20	2	0		2	93		90	3	1			1
20	96	46	18/1/21	7	7		0	89		86	3				
21	96	46	18/1/21	5	5		0	91		85	6				
22	96	46	18/1/21	6	2		4	90		88	2				
23	372	186	25/1/21	19	11		8	351		333	18	2		1	1
24	96	46	18/1/21	4	4		0	92		92	0				
25	96	46	18/1/21	5	5		0	91		91	0				
26	96	46	18/1/21	6	3		3	90		90	0				
27	96	46	21/1/21	6	4		2	90		90	0				
28	96	46	26/1/21	6	4		2	90		79	11				
29	96	46	26/1/21	3	2		1	93		83	10				
30	96	46	4/2/21	6	4		2	90		88	2				
31	96	46	4/2/21	3	1		2	92		87	5	1			1
32	96	46	4/2/21	5	5		0	90		89	1	1			1
33	96	46	7/2/21	6	6		0	90		88	2				
34	96	46	7/2/21	6	6		0	90		89	1				
35	96	46	7/2/21	6	2		4	90		82	8				
**Total**	**3636**	**1351**		**141**	**97**	**1**	**43**	**3482**	**0**	**3383**	**99**	**13**	**0**	**5**	**8**

Supplementary Table 2: Summary of side-by-side data

Pooling Design (#samples to #pools)	Efficiency	disease prevalence	Number of experiments
96 to 25	3.8	2	19
96 to 46	2.1	6	15
372 to 186	2	8	1
