# Supplementary material for: High capacity clinical SARS-CoV-2 molecular testing using combinatorial pooling
Source: Commun Med (Lond). 2024 Jun 19;4:121. doi: 10.1038/s43856-024-00531-w (PMC11187214; doi:10.1038/s43856-024-00531-w)
Supplement: Supplementary file 3 — Description of Additional Supplementary Files [file 43856_2024_531_MOESM3_ESM.pdf]

Description of Additional Supplementary Files

Supplementary Data 1: Source data underlying the figure 2a.

Supplementary Data 2: Source data underlying the figure 2b.

Supplementary Data 3: Source data underlying the figure 3a.

Supplementary Data 4: Source data underlying the figure 3b.

Supplementary Data 5: Source data underlying the figure 3c.

Supplementary Data 6: Source data underlying the figure 3d.

Supplementary Data 7: Results of a pilot experiment of multiplex PCR pooling using a pooling design that tests 96 samples in 46 wells. Pools and Individual samples were tested using the Seegene Allplex™ RV7 commercial PCR kit. Each row corresponds to a single individual (n=96). Positive detections using individual sample testing are denoted by the Ct value of the RV7 kit. Colors denote samples identified by P-BEST: Green – sample classified as ‘positive’; Yellow – sample classified as ‘suspected’.

Supplementary Data 8: Results of a pilot experiment of multiplex PCR pooling using a pooling design that tests 96 samples in 46 wells. Pools and Individual samples were tested using the GeneXpert FLU/RSV commercial PCR kit. Each row corresponds to a single individual (n=96). Positive detections using individual sample testing are denoted by the Ct value of the GeneXpert kit. Colors denote samples identified by P-BEST: Green – sample classified as ‘positive’; Yellow – sample classified as ‘suspected’.

Supplementary Data 9: Results of a pilot experiment of multiplex PCR pooling using a pooling design that tests 96 samples in 25 wells. Pools and Individual samples were tested using the GeneXpert RSV/FLU commercial PCR kit. Each row corresponds to a single individual (n=96). Positive detections using individual sample testing are denoted by the Ct value of the GeneXpert kit. Colors denote samples identified by P-BEST: Green – sample classified as ‘positive’; Yellow – sample classified as ‘suspected’.
